# Identification, cloning and functional characterization of novel sperm associated antigen 11 (SPAG11) isoforms in the rat

**DOI:** 10.1186/1477-7827-4-23

**Published:** 2006-04-28

**Authors:** Suresh Yenugu, Katherine G Hamil, Gail Grossman, Peter Petrusz, Frank S French, Susan H Hall

**Affiliations:** 1 Laboratories for Reproductive Biology, Department of Pediatrics, University of North Carolina, Chapel Hill, NC 27599-7500, USA; 2 Department of Cell and Developmental Biology, University of North Carolina, Chapel Hill, NC 27599-7500, USA; 3 Department of Biochemistry and Molecular Biology, Pondicherry University, Pondicherry, 605014, India

## Abstract

**Background:**

Sperm binding proteins and their C-terminal peptides of the Sperm Associated Antigen 11 (SPAG11) family were found to play an important role in epididymal innate immunity in addition to their role in sperm maturation. However, the expression of Spag11 transcripts in rodents is not well documented.

**Methods:**

Computational analysis was employed to identify novel Spag11 isoforms in the rat. RT-PCR analyses were carried out on RNAs isolated from the male reproductive tract tissues of rat using gene specific primers for Spag11c and Spag11t. The identities of PCR products were confirmed by sequencing. Tissue distribution, developmental expression and androgen regulation of Spag11t and Spag11c were studied using RT-PCR. The antimicrobial activities of recombinant Spag11t and Spag11c were tested against E coli in a colony forming unit assay.

**Results:**

In this study, we identified two novel Spag11 transcripts, namely, Spag11t and Spag11c derived from the long arm of chromosome 16 in the rat (Rattus norvegicus), using both in silico and molecular biology approaches. Spag11c is expressed in all three regions of the epididymis, in testis and in ovary but is absent from the seminal vesicle. Spag11t expression is confined to the caput and it is not expressed in the testis, seminal vesicle or ovary. Age dependent expression of Spag11t and Spag11c was observed in the epididymides of rats (10–60 day old). Their expression was found to be most abundant in the adult rat (60 day) suggesting roles in mature reproductive function. Further, both Spag11t and Spag11c expression was down regulated in castrated rat epididymides and the expression was maintained in the testosterone replaced castrated rats. SPAG11C is a potent antibacterial agent. SPAG11T also displayed bactericidal capacity although weaker than SPAG11C and SPAG11E.

**Conclusion:**

The abundant expression of Spag11t and Spag11c in the male reproductive tract suggests an important role in male reproductive tract immunity. Their expression is developmentally regulated and androgen dependent. Characterization of novel SPAG11 isoforms will contribute to our understanding of the role of epididymal proteins in sperm maturation and innate immunity.

## Introduction

Spermatozoa leaving the testis are immature and lack fertilizing ability. During their passage through the epididymis, which provides the environment for sperm maturation and storage, the spermatozoa acquire fertilizing ability and forward motility after interacting with proteins secreted by the epididymal epithelial cells. These secreted proteins are thought to be involved in a range of general and specific reproductive activities including the initiation of sperm maturation [1], sperm-oocyte recognition [2] and the acrosome reaction [3]. Recent evidence suggests that epididymal antimicrobial proteins including defensins [4-7], SPAG11 isoforms A, D, E and G [8,9], protease inhibitors [10-13], cathelicidins [14] and other proteins [15,16] function in male reproductive tract immunity as well.

Defensin gene identification and expression analyses in the male tract of different species have driven efforts to understand defensin evolution and function in part because of their potential to control the spread of sexually transmitted diseases [17]. The more than 30 beta-defensin genes identified in humans are organized in five chromosomal regions [18]. In humans, the *SPAG11 *gene (also known as *human epididymal protein 2 *(*HE2*) and *epididymal protein 2 *(*EP2*)), is located within the β-defensin gene cluster on chromosome 8p23 [19]. This *SPAG11 *transcription unit evolved from the fusion of 2 ancestral β-defensin genes designated the A and B components of *SPAG11 *[20]. Human and monkey *SPAG11 *transcripts are alternatively spliced to encode at least 19 proteins in which different exon-encoded modules are variously combined [21]. Alternative splicing of primate *SPAG11 *transcripts is differentially regulated in different male organs in different species [21], possibly a response to preferential colonization of different organs by different pathogens [22,23]. Primate SPAG11 mechanisms of antibacterial action have been investigated in detail [8,9,21]. Primate *SPAG11 *gene expression is regulated by testicular androgen [24], a major effector molecule driving gene expression, protein synthesis and secretion in the epididymis [25,26]. However, the expression of *Spag11 *variants in rodents is less well understood. Only a single rodent isoform has been well characterized, SPAG11E, product of a gene orthologous to the B component [27] of the primate *SPAG11 *fusion gene. In mouse [7] and rat, *Spag11e *(known in rat as *Bin1B*) [28] expression was reported to be epididymis-specific and the protein contains the defensin-like 6 cysteine motif. Antibacterial activity was demonstrated for rat SPAG11E [28] which is androgen-regulated [27] and has been implicated in the initiation of sperm maturation [1]. Dual roles in host defense and sperm maturation suggest that the multifunctionality that characterizes other defensins [29] is also true of epididymal defensins.

Despite these extensive studies on rodent *Spag11e *and a recent report mentioning *Spag11c *[30], orthologs of the other 19 *Spag11 *transcripts found in primates have not been described in the rodent. In this report, rat *Spag11 *component A [27] is characterized revealing the transcription of novel alternatively spliced mRNA *Spag11t *in addition to *Spag11c*. The expression of these transcripts in epididymis is age and androgen dependent. SPAG11C and T proteins are demonstrated to be potent antibacterial agents.

## Materials and methods

### Genomics

Using sequence from mouse *ab initio *SPAG11 model hmm21500, LOC546038 to search rat genome using the BLAST program at the NCBI website , rat gene model hmm24581 was identified as the rat SPAG11 A component. Intron spanning primers (Table 1) were designed and RT-PCR performed using rat epididymis mRNA as the template. The specific products were sequenced and deposited in Genbank. The corresponding exon/intron boundaries were determined by aligning the cDNA with the genomic sequences and were deposited in Genbank. The sequences were translated in all six reading frames using the ExPASy website .

**Table 1:** Gene specific primer sequences for *Spag11t *and *Spag11c*

***Gene***	**Primer sequence**
***Spag11t***	**Forward – 5' CTG CAG TCC CCT CCA CAG CC 3'** **Reverse – 5' CAT CCA CGC TGT CAC CTC CC 3'**
***Spag11c***	**Forward **– **5' CTG CAG TCC CCT CCA CAG CC 3'** **Reverse **– **5' GTG TGC AGG TCA CTT CAA CTT C 3'**

### Tissue specimens and RT-PCR

Wistar rat (aged 60–90 days) tissues were obtained commercially (Zivic Laboratories Inc, Pittsburgh, PA, USA). Prior to shipping on dry ice, tissues were placed in RNA*Later *(Ambion, Inc Austin TX, USA) solution overnight at 4°C to allow penetration and fixation. Upon arrival, tissues were immediately stored at -70°C. Total RNA was extracted using the TRIzol reagent (Invitrogen, Carlsbad, CA, USA) from the following tissues: caput, corpus, cauda, testis, seminal vesicle, prostate, spleen, heart, lung, liver and kidney from a single adult male and ovary, uterus, mammary gland and cervix from a single adult female. Total RNA (2 μg) was reverse transcribed using 50 U Stratascript (Stratagene, La Jolla, CA, USA) and 0.5 μg of oligodT (Invitrogen) according to the manufacturer's instructions. 2 μl of the resultant cDNA was amplified by PCR using gene specific primers (Table 1). The number of cycles to amplify each cDNA in the linear range was determined by preliminary PCR under the following conditions: 94°C for 1 min, followed by 25–35 cycles at 94°C for 30 sec, 56°C for 30 sec and 72°C for 30 sec, and with a final round of extension at 72°C for 10 min. *Spag11c *and *Spag11t *were amplified for 32 cycles and *Gapdh *for 28 cycles. PCR amplified gene products were analyzed by electrophoresis on 2 % agarose gel. Identity of major amplicons was determined by sequencing at the UNC-CH Genome Analysis Facility using ABI PRISM model 377 DNA sequencer (PE Applied Biosystems, Foster City, CA, USA). Glyceraldehyde phosphate dehydrogenase (*Gapdh*) expression was used as the internal control. To study the androgen regulation of *Spag11 *transcripts, epididymides from sham operated, castrated and testosterone supplemented Sprague-Dawley (aged 120–150 days) rats were obtained from Dr. Christopher Wingard (Brody School of Medicine, Greenville, NC, USA). Briefly, five rats were sham operated and ten were castrated. Immediately after castration, five rats received 20 mg dihydrotestosterone implants. All the animals were sacrificed 14 days after castration. Whole epididymis were collected in RNA*Later *and processed as described above. All procedures were performed in accordance with the Guiding Principles in the Care and Use of Animals established by the National Institute of Health and approved by the Institutional Committee on the use of Animals in Research and Education. For studies on the developmental regulation of *Spag11*, epididymides from 10–60 day old Wistar rats, one rat of each age, were obtained commercially (Zivic Laboratories).

### Antibody production

Peptides were synthesized using a Rainin Symphony multiple peptide synthesizer (Rainin Instruments, Woburn, MA) using fluorenylmethyloxycarbonyl chemistry in the University of North Carolina Program in Molecular Biology Protein Chemistry Facility. Peptides were purified by HPLC. Peptide sequences were based on mouse SPAG11C (YQIVNSKKSEGQSQEC), and SPAG11T (KSSIQDQSKFIHLGNC) in which the internal cysteines were replaced with serines to minimize disulfide bond-mediated aggregation. These mouse peptides are very similar to the rat sequences in these regions. C-terminal cysteines were conjugated to keyhole limpet hemocyanin. Antibodies were raised in rabbits at Bethyl Laboratories, Inc (Montgomery, TX). Antibodies were affinity purified on peptide antigen columns prepared using the Sulfo-Link kit (Pierce, Rockford, IL) according to the manufacturer's recommendations. The resulting antibodies cross react well with the rat proteins.

### Immunostaining

Adult Sprague-Dawley rat epididymides were fixed in Bouin's fluid and embedded in paraffin. Sections were immunostained using a Vectastain Standard ABC kit (avidin-biotin-complex horse radish peroxidase) (Vector Laboratories Inc., Burlingame, CA, USA). Diaminobenzidine, the chromogen, produced a brown reaction product. Sections were counterstained with toluidine blue. For the control staining, antibodies were preincubated with antigen peptide. Photographs were taken using a SPOT Cooled Color digital imaging system (Diagnostic Instruments, Inc, Sterling Heights, MI, USA) attached to a Zeiss Photomicroscope III. Photographs were prepared using SPOT image processing software. Images were arranged using PhotoShop (Adobe Systems Inc, San Jose, CA, USA).

### Recombinant protein production

Recombinant proteins were prepared as described earlier [8]. Open reading frame that correspond to the rat SPAG11T or SPAG11C or SPAG11E without the signal peptide (amino acid sequence shown in italics in Figure 2), defined as the mature protein in this study, was cloned into pQE80 expression vector (Qiagen, Valencia, CA). *E. coli *(OrigamiB (DE3) pLacI^q ^was transformed with the pQE80 vector containing rat *Spag11t *or *Spag11c *or *Spag11e *cDNA according to the supplier's instructions. Transformed *E. coli *were grown to mid-log phase and fusion protein expression was induced with 1 mM isopropyl-1-thio-β-D-galactoside for 1 h at 37 C. To avoid baseline expression of the protein prior to induction, 1% glucose was maintained in the bacterial medium and the induction time was kept to a minimum (1 h) to minimize the toxic effects of the peptides on *E. coli*. Bacterial lysate was incubated with nickel-nitrilotriacetic acid-agarose (Qiagen, Valencia, CA) for 1 hour at room temperature to allow binding of His-tagged recombinant protein to the resin. It was then transferred to a column, washed and the recombinant protein eluted according to the manufacturer's recommendations. Fractions were analyzed on 10–20% gradient polyacrylamide Tris-Tricine gels and stained with Coomassie blue G250. Fractions containing purified protein were pooled and dialyzed against 10 mM sodium phosphate buffer (pH 7.4) to remove urea. The His-tagged recombinant SPAG11 proteins contained the following additional amino acid residues at their N-termini (MRGSHHHHHHGS) due to the construction of the vector.

**Figure 1:**
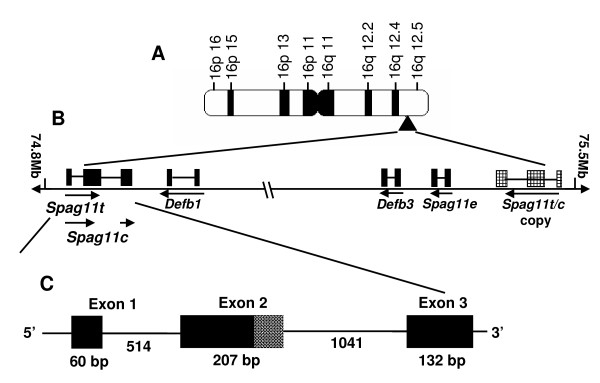
Rat *Spag11t/Spag11c *localization on Chromosome 16. **A**, Ideogram of rat Chromosome 16. **B**, Arrangement of genes from 75.5 megabases to 74.8 megabases (not to scale). Arrows indicate direction of transcription. Positions were taken from the MapView (build 3.1) at The National Center for Biotechnology Information (NCBI) website. **C**, Intron-Exon structure of rat *Spag11t/Spag11c *gene. Black boxes indicate translated regions of the exons. The region of exon 2 not included in *Spag11c *after the intrinsic splice site is shown as a hashed box.

**Figure 2:**
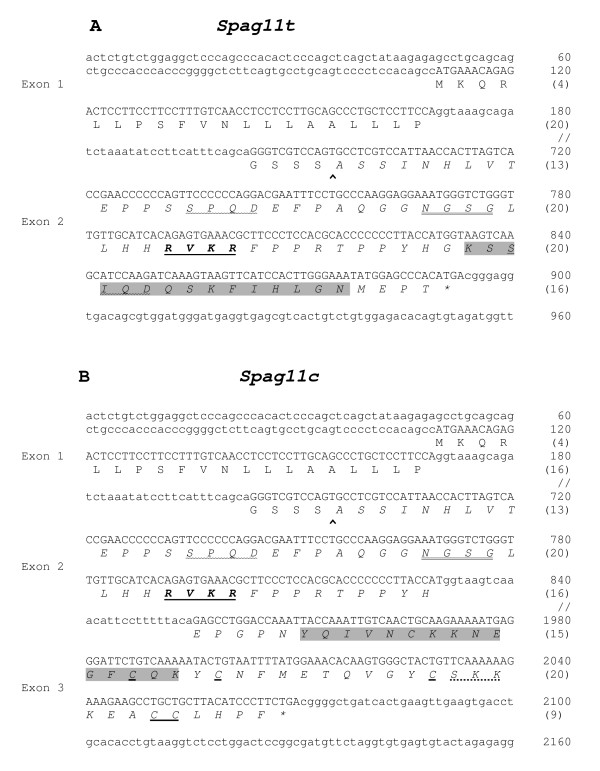
Rat chromosomal sequence aligned with *Spag11t *(A) and *Spag11c *(B) mRNA and amino acid sequences. Exons are in upper case letters, introns in lower case. Amino acids are indicated in single letters. Numbers in parentheses indicate amino acids of the protein. The gene sequence was extracted from Genbank NW_047475. The rat cDNA sequences for *Spag11t *and *Spag11c *are available at Genbank and were assigned the accession numbers AY600144 and AY600145. ^ indicates predicted signal peptide cleavage site. Cysteines are bold underlined. Amino acid sequence shown in italics was cloned and expressed to test their antimicrobial activity. The antibodies were raised against the mouse peptides corresponding to the indicated shaded regions. Furin-like proprotein convertase motif is in bold and underlined. Posttranslational modification sites are indicated: double underlined – N-glycosylation; wavy underlined – casein kinase II phosphorylation; dotted underlined – protein kinase C phosphorylation.

### Antibacterial assays

Colony forming units (CFU) assay was employed to test the antibacterial activity as described earlier [8]. *E. coli *was used to test the activity since it is one of the common causative agents of epididymitis. Briefly, overnight cultures of *E. coli *XL-1 blue (Stratagene, La Jolla, CA) allowed to grow to mid-log phase (A_600 _= 0.4 - 0.5) were diluted with 10 mM sodium phosphate buffer (pH 7.4). Approximately 2 × 10^6 ^CFU/ml of bacteria were incubated at 37 C with 1–10 μM of SPAG11 proteins for 0–120 min. Aliquots of the assay mixture taken out at 30, 60 and 120 min after incubation were serially diluted with 10 mM sodium phosphate buffer (pH 7.4) and 100 μl of each was spread on a LB agar plate and incubated at 37 C overnight to allow full colony development. The resulting colonies were hand counted and bacterial survival expressed as CFU/ml.

## Results

Two copies of the *Spag11 *A component encoding *Spag11c *were discovered in a β-defensin-rich region of rat chromosome 16q12.5 using a homology search strategy. One copy of the *Spag11c *gene located adjacent to the *Spag11e *gene (B component) is oriented in the same direction (Fig 1). The second gene copy is located more than 0.6 Mb away from the *Spag11e *gene (Fig. 1). The distant *Spag11 *A component is located within a genomic segment that also includes a copy of *ab initio *gene model hmm24586 and thus is at least 250 kb long. The segment appears to be copied from a region near the *Spag11e *gene and inverted when inserted into its new site. The inversion results in transcription in the opposite direction from the *Spag11e *gene precluding the read-through transcription that in primates creates functional fusion of the two ancestral β-defensin genes [31]. Our efforts and those of Patil *et al*. [30] to detect the read-through transcripts, *Spag11d *or *Spag11q *failed (data not shown). Since these could only be initiated at the adjacent *Spag11 *A component, their absence is consistent with low transcriptional activity of the adjacent copy. Our *Spag11c *cDNA sequence obtained by RT-PCR exactly matches the distant copy, but is only 97% identical to the adjacent gene copy also suggesting the distant copy is more transcriptionally active. This result is similar to previously reported unequal transcriptional activity among duplicated human *SPAG11 *loci [32].

Expressed sequence analysis revealed the *Spag11c *transcript contains exon 1 sequence encoding the predicted signal peptide spliced to exon 2 sequence which contains a conditional splice site at which it is joined to the exon 3-derived portion of the transcript (Fig. 1). In addition, this study identified a novel transcript we named, *Spag11t*. In the *Spag11t *transcript, the splice site inside exon 2 is ignored. Thus, the *Spag11c *and *Spag11t *transcripts encode the identical signal peptide suggesting both are secreted as well as the identical N-terminal peptide. In dissimilar C-terminal peptides, SPAG11C contains a 6-cysteine defensin motif whereas SPAG11T contains no recognized antibacterial motif. Further, the well characterized furin-like proprotein convertase motif (RVKR, amino acids (aa) 33–36) reported earlier in human SPAG11 isoforms [33] was also identified (Fig. 2). A PROSITE [34] scan identified several consensus post-translational modification sites including an N-glycosylation site (NGSG, amino acids (aa) 25–28), casein kinase II phosphorylation sites (SPQD, aa 14–17; and SIQD, aa 49–52 in SPAG11T only) and a protein kinase C phosphorylation site in SPAG11C (SKK, aa 78–80) (Fig. 2). Other general characteristic features of SPAG11T and SPAG11C are described in Table 2.

**Table 2:** General characteristic features of rat SPAG11 protein isoforms

	**SPAG11T**	**SPAG11C**
**Length**^a^**(aa)**	**65**	**89**
**MW (kD)**^a^	**10.2**	**7.3**
**pI**^a^	**9.52**	**8.82**
**Cysteines**^b^	**0**	**6**
**Net Charge**^a^	**+1**	**+3**
**Identity**^c^	**49% 47–aa**	**58% 88-aa**

^a ^amino acids in mature protein not including the signal peptide

^b ^number of cysteines in the C termini

^c ^Identity indicates percent identical amino acids over the indicated length in humans

**Figure 3:**
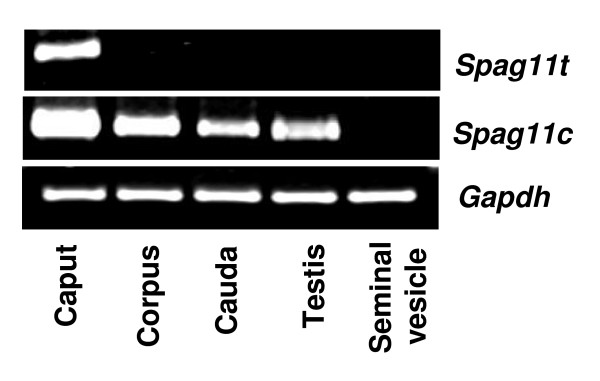
Rat *Spag11 *expression in male reproductive tissues. Total RNA isolated from caput, corpus, cauda, testis and seminal vesicle was reverse transcribed and amplified by PCR.

**Figure 4:**
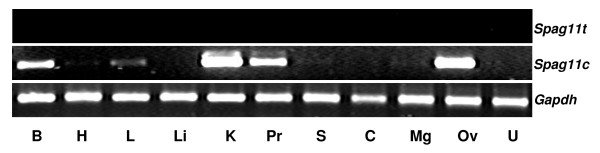
Expression of rat *Spag11 *in different tissues. RT-PCR analysis of *Spag11t *and *Spag11c *gene expression in RNA isolated from **B**rain, **H**eart, **L**ung, **Li**ver, **K**idney, **P**rostate, **S**pleen, **C**ervix, **M**ammary **g**land, **Ov**ary, **U**terus. *Gapdh *was used as the internal control.

To expand our understanding of the potential functions of rat SPAG11 isoforms, we investigated the variant RNA expression in different organ systems. In the male reproductive tract, *Spag11t *was confined to the caput region in the epididymis and was absent from testis and seminal vesicle (Fig. 3) and was also not expressed in any of the 11 other tissues analyzed (Fig. 4). *Spag11c *was expressed in epididymis and testis (Fig. 3). It was also expressed in brain, lung, kidney, prostate and ovary (Fig. 4). Thus although the A component of the *Spag11 *gene was transcribed in many tissues, only in the caput was the internal splice site of exon 2 ignored allowing formation of the *Spag11t *mRNA. This result suggests highly specific mRNA splicing mechanisms in caput and is consistent with highly specific function for the SPAG11T protein in caput perhaps a role in sperm maturation. The broader distribution of SPAG11C may reflect evolution away from a male-specific function and perhaps an antibacterial activity that functions well in the environments of different organs.

**Figure 5:**
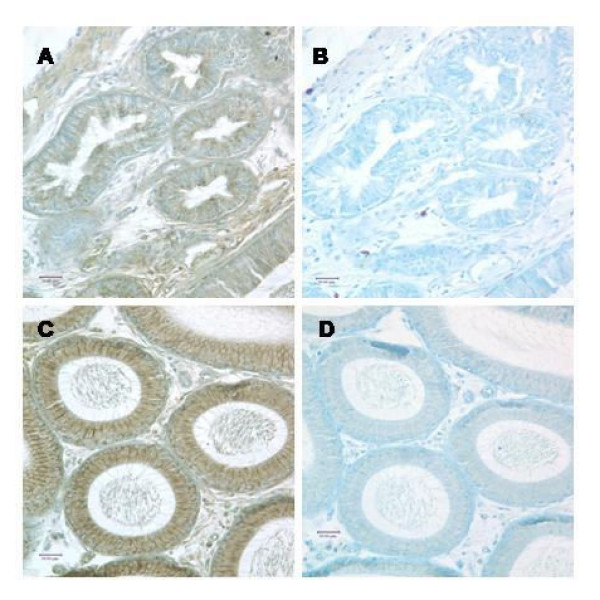
Immunolocalization of rat SPAG11T. A, Efferent ducts; C, Caput; B and D serial sections using antibody preadsorbed with peptide antigen. Antibody/antigen peptide ratio: 8 μg/ml/100 μg/ml.

**Figure 6:**
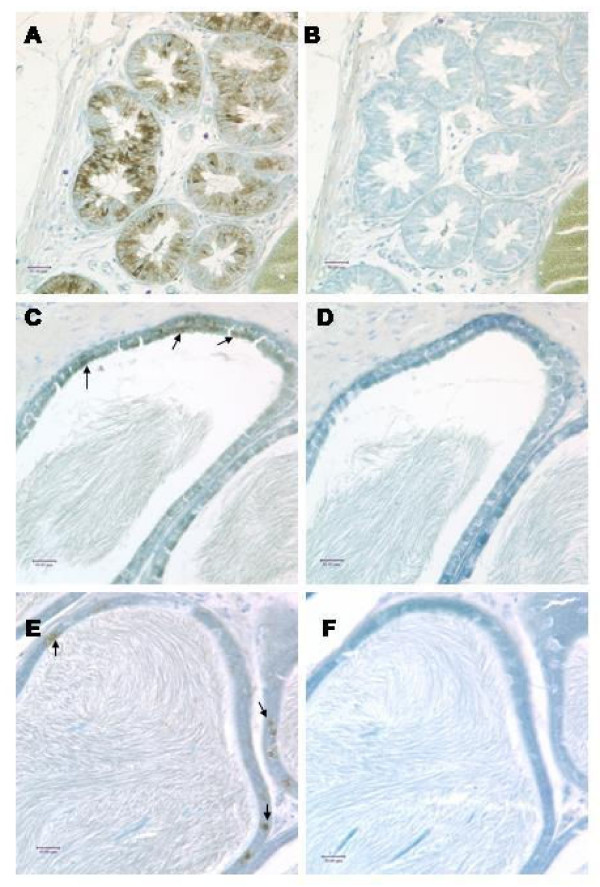
Immunolocalization of rat SPAG11C. A, Efferent ducts; B, Corpus; C, Cauda; B, and D and F serial sections using antibody preadsorbed with peptide antigen. Arrows indicate SPAG11C staining which appears brown against the toluidine blue counter stain. Antibody/antigen peptide ratio: SPAG11C – 2 μg/ml/100 μg/ml in epididymis.

**Figure 7:**
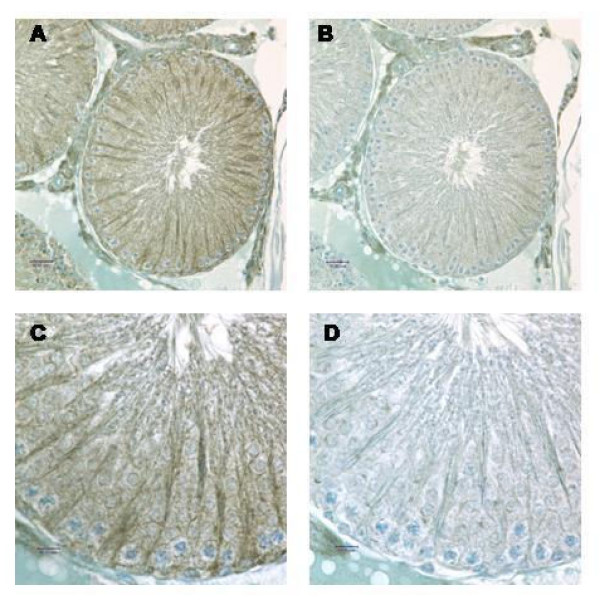
Rat SPAG11C immunolocalization in the testis. A, taken with 40X objective; C, 100X objective. B and D serial section using antibody preadsorbed with peptide antigen. Antibody/antigen peptide ratio: 8 μg/ml/100 μg/ml.

These tissues were immunostained to confirm protein expression. SPAG11T protein is abundant in initial segment and caput (Fig. 5). Stronger staining of SPAG11C protein was observed in a banded pattern in the principal cells of the efferent duct epithelium, corpus and cauda (Fig. 6), but weaker staining was seen in the initial segment and caput epididymis (data not shown). In testis, SPAG11C staining was detected throughout the germinal epithelium and appeared especially strong in Sertoli cells (Fig. 7).

**Figure 8:**
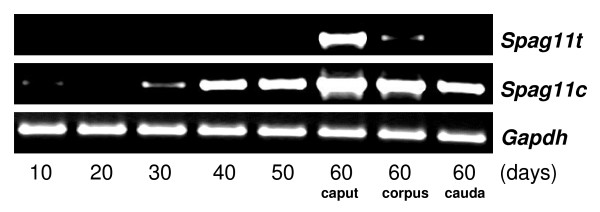
Age dependent expression of rat *Spag11*. RT-PCR for *Spag11t *and *Spag11c *in RNA isolated from epididymis of rats aged 10–60 days. *Gapdh *was used as internal control.

**Figure 9:**
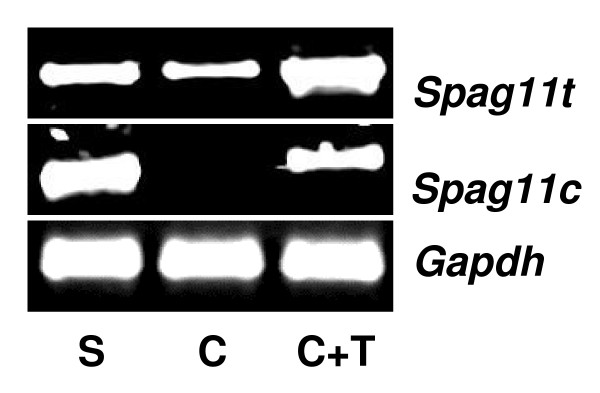
Androgen regulation of *Spag11 *expression. Rats (n = 5 for each group) were sham operated (S), castrated (C), or castrated and testosterone replaced immediately after castration (C+T). Epididymides were removed 14 days after castration. Gene expression was analyzed using RT-PCR with *Gapdh *as the internal control.

As a first step toward determining if androgen regulates expression of *Spag11c *and *Spag11t *as previously shown for *Spag11e *[28], age-dependent expression was analyzed in 10 to 60 day old rats (Fig. 8). *Spag11t *mRNA was present exclusively in adult rats where high levels of testosterone maintain the fully functional epididymis. Early onset of *Spag11c *expression in prepubertal rats when testosterone levels are low raises the possibility that other factors may be involved in regulating the expression of these splicing variants. Moreover, although the *Spag11 *A component appears transcriptionally active in the prepubertal rat producing the *Spag11c *mRNA, the co-transcriptional splicing mechanisms that produce the *Spag11t *mRNA variant are not established until adulthood when the *Spag11t *splicing is almost entirely restricted to the caput. Next, the effects of androgen ablation and replacement on *Spag11 *expression were investigated in the epididymides of rats that were sham operated, castrated and castrated with immediate testosterone replacement (Fig. 9). In the castrated animals, expression levels of *Spag11t *were somewhat suppressed and *Spag11c *expression was abolished. Thus, in the castrated rats, transcription rates and/or mRNA stability were reduced for products of both *Spag11 *A and B promoters, but the more dramatic effects are on the A promoter-dependent *Spag11c *mRNA, suggesting androgen effects on both mRNA levels and mRNA splicing. Testosterone replacement maintained the expression of these mRNAs confirming androgen-dependent expression.

**Figure 10:**
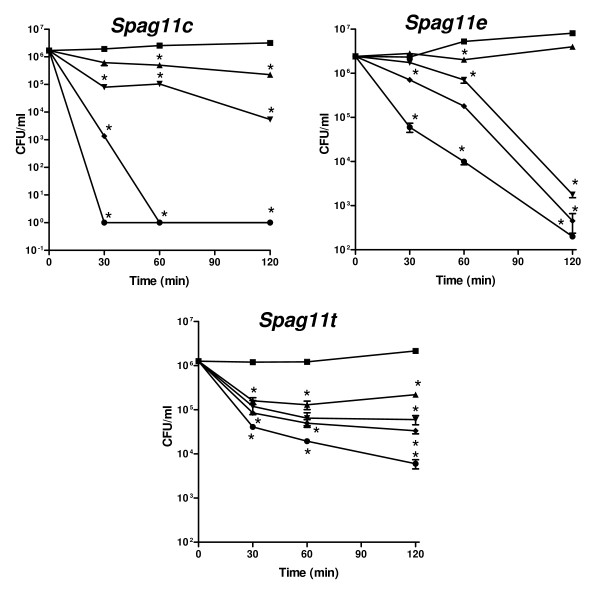
Kinetics of bacterial killing by rat SPAG11 isoforms. Mid-log phase *E. coli *were incubated with 1–10 μM of SPAG11 protein for 0–120 min. (■) 0 μM; (▲) 1 μM; (▼) 2μM; (◆) 5 μM; (●) 10 μM. Data (Mean ± S.E) shown are representative of three independent experiments. *p < 0.001 when compared to 0 μg/ml.

Unlike the direct quantitative antibacterial analyses of human and primate SPAG11 isoform activities [8,21], in rat, the SPAG11E protein antibacterial activity was indirectly assessed by overgrowth of bacteria after suppression of the *Spag11e *mRNA [28]. To obtain direct evidence of the antibacterial activity of rat SPAG11 isoforms, *E. coli *were exposed to increasing amounts of these proteins for up to 2 h. The defensin-like C isoform exhibited potent dose and time-dependent bacterial killing activity followed by the SPAG11E isoform, which also contains the 6-cysteine array (Fig. 10). The T isoform, which contains no known antibacterial motifs, was the least potent of the three proteins (Fig. 10). In our previous studies, the epididymal lipocalin and an unrelated protein, bovine serum albumin were included as negative controls in the antibacterial assays. These proteins did not exhibit any antibacterial activity [8].

## Discussion

Emerging from mammalian genomic analyses are major new insights into the evolution and function of innate immunity genes [32,35-38]. Our current investigations into the rat *Spag11 *genes led to the discovery of 2 copies of rat *Spag11 *gene A component, one adjacent to and one displaced 0.6 Mb from the established B component. Conservation of both components in rodents and primates since their divergence 80 million years ago affirms their essential contribution to the fitness of these mammalian species. Our demonstration of antibacterial activity of the SPAG11C and T isoforms supports their function in innate immunity and their abundant expression in epididymal epithelium is consistent with a role in sperm maturation.

The distant physical separation and apparently obligate autonomous functioning of the A and B components of rat *Spag11 *depart from the fusion gene paradigm of primate *SPAG11 *and suggest the rat gene is evolving in a unique direction at this level. In primates, fusion transcripts encompassing A and B components are the predominant *SPAG11 *transcripts [39]. In human genome build 35.1, the two copies of the *SPAG11 *gene (Hs.2717 and Hs.459418 at 7.3Mb and 7.75 Mb) both contain A and B components and ESTs suggest read-through transcription of both copies. Our results indicate the distant *Spag11c *gene is transcriptionally dominant whereas Patil *et al*. [30] reported transcriptional activity at the adjacent copy. The reason for this difference is not known.

Inversion polymorphisms in the β-defensin-rich region of human chromosome 8p23.1 containing the *SPAG11 *fusion gene are common events, present in 25% of the normal population and as many as 12 *SPAG11 *paralogs may reside in an individual genome [32,40,41]. Duplication of antibacterial genes suggests potential for enhanced infectious disease resistance in these individuals [32], although data supporting health correlates of gene copy number are still lacking for β-defensins. In fact, mRNA levels for neighboring polymorphic genes in 8p23.1, *DEFBA1 *and *DEFBA3 *fail to correlate with gene copy number [42]. However a positive correlation was demonstrated for a chemokine gene cluster containing variable copy numbers and expression levels and a link to individual resistance to pathological progression was suggested [43]. Additional analyses of the rat genome are needed to determine whether the chromosomal region in rat orthologous to human 8p23 is similarly structurally dynamic, perhaps positively contributing to the well-known fitness of this troublesome species.

High sequence identity of the two A component copies suggests the duplication event was recent. Recent duplication and translocation are also suggested by the absence of the distant copy from the mouse genome which diverged from rat about 40 million years ago [44]. This recent duplication is evidence of active genomic evolution and may be related to adaptation of the rat to changing environments [45]. The absence from mouse follows the general trend of lower incidence of high identity duplicated sequence within the mouse genome (1%-1.2%) compared to the rat assembly (2.92%) which is lower than the duplicate frequency in human (5%) [45].

Segmental expression analysis revealing that rat *Spag11 *variant transcripts and proteins in epididymis are most abundant in the caput region is similar to expression reported for primate *SPAG11 *[24,46,47]. Our study shows that, unlike primate *SPAG11 *variants and other defensins which are predominantly expressed in the male reproductive tract [21,35], rat *Spag11c *is expressed in the female and also in several non-reproductive tissues in the male suggesting broader molecular function than the primate forms. Its localization in the testis and epididymis suggests that it could act as an antibacterial protein in both organs. Further, its localization in the seminiferous tubules of the testis could suggest a role in spermatogenesis. Though most studies propose the involvement of epididymal proteins in sperm maturation, a role for SPAG11 proteins in spermatogenesis is not investigated. Studies addressing this angle may define a broader role for SPAG11 proteins and peptides beyond sperm maturation and male reproductive tract immunity. Further, we show that the three rat SPAG11 mature protein isoforms without the signal peptide do maintain functional congruence with human primate SPAG11 in direct killing of bacteria. It is interesting to note that though SPAG11C exhibits more potent antibacterial activity than SPAG11T and E isoforms, its pI (8.82) is less than that of SPAG11T (9.52) and SPAG11E (9.43). Structural factors besides the general physical parameters are thought to influence the activity of antimicrobial proteins and peptides. For example, the platypus defensin-like protein, DLP-3 (pI 8.5, net charge +2) and the C-terminal peptide of hSPAG11C which lack amphipathic transitions and the DLPs -1 (8.33, +2) and -2 (7.77, +1), which have amphipathic transitions are all inactive [48,49].

In humans, SPAG11 protein isoforms contain a furin-like proprotein convertase motif [33,50]. SPAG11 peptides resulting due to cleavage at the furin-like proprotein convertase motif are reported in the epididymal fluid, ejaculate and on sperm and their antimicrobial activity demonstrated [33]. Protein sequence analysis in this study reveals the presence of furin-like proprotein convertase motif (RVKR) in rat SPAG11C and T. Conservation of this recognition site in rat SPAG11 suggests the existence of a similar processing mechanism in rodents and the existence of SPAG11 peptides generated due to this processing mechanism is a definite possibility. Although the DLLP-peptide appears to be the dominant form in human epididymal fluid and seminal plasma, the presence of full length proteins that we have tested in the present study cannot be ruled out. Further experiments will be needed to determine the proteolytic processing of rat SPAG11 isoforms at the furin-like proprotein convertase motif and their existence in the epididymis, whether the processing occurs primarily inside the epithelium or after secretion into the lumen, whether a fraction or all of the SPAG11 isoforms secreted are cleaved at the furin-like proprotein convertase motif and to define which forms achieve effective bactericidal concentrations in the lumen. The functional consequence of cleavage by furin is to release the active SPAG11A and SPAG11D antibacterial C-terminal peptides and the SPAG11C peptide that is inactive against bacteria [33,50]. The N-terminal 35 amino acid peptide that is simultaneously generated by furin cleavage may exhibit antibacterial activity as well since the N-terminal 46 amino acid peptide is an effective bactericide [51]. It will be important in future studies to compare these two N-terminal peptides in the same experiments to determine if the additional amino acids quantitatively affect the antibacterial activity.

Expression of *Spag11c *in the 30 day rat somewhat in advance of the rapid increase in circulating testosterone levels that occurs days 33–55 [52] may be an indication that additional factors stimulate the onset of prepubertal expression. On the other hand, *Spag11t *expression only in the adult rats suggests that its expression is primarily dependent on testosterone levels and may have a role in maturation and spermatogenesis. It is interesting to note that in the 60–90 day old rats, *Spag11t *was restricted only to the caput (Figure 3), whereas there was some expression in the 60 day old rat corpus (Figure 8). This discrepancy could be due to variations in the age of the animals used. In addition to androgens, testicular factors including basic fibroblast growth factor and androgen binding protein are known to affect epididymal gene expression [25,26]. However in the adult rat, dramatic reduction in expression levels after castration and maintenance of expression by testosterone replacement indicate regulation of these variants may primarily depend on androgen. Similar results were reported for the rat *Spag11e *variant [27] and for collective *SPAG11 *transcripts initiated at the A component in primate [24].

We show that an important and conserved role for the SPAG11C and T protein isoforms lies in host defense of the male reproductive tract. Furthermore, our data showing developmental and androgen regulation of rat *Spag11c *and *Spag11t *in epididymis implicate these proteins in mature epididymal function. Reports showing rat SPAG11E roles in host defense [28] and initiation of sperm maturation [1] support this hypothesis for the E isoform and suggest that other isoforms should be tested for male-specific functions as well. The antibacterial mechanism of SPAG11C and T may be due to their interaction with the negatively charged bacterial membranes thus facilitating bacterial membrane permeabilization. Such a mechanism of antibacterial action was earlier demonstrated for human and macaque SPAG11 protein isoforms [8,53]. Our data particularly highlight the SPAG11T isoform for potential function in sperm maturation as its expression was not detected outside the caput epididymis where sperm maturation is the primary function.

## Authors' contributions

SY performed the *in silico *analysis, PCRs, recombinant protein expression, antibacterial assays and wrote majority of the manuscript. KGH cloned *Spag11c *and *Spag11t*. GG and PP performed and analyzed the immunohistochemical staining. SHH and FSF supervised and coordinated the work and the preparation of the manuscript. All authors read, commented upon and approved the final manuscript.
